# Models, Regulations, and Functions of Microtubule Severing by Katanin

**DOI:** 10.5402/2012/596289

**Published:** 2012-09-27

**Authors:** Debasish Kumar Ghosh, Debdeep Dasgupta, Abhishek Guha

**Affiliations:** Department of Biological Sciences, Indian Institute of Science Education and Research, Kolkata, Mohanpur, Nadia 741252, India

## Abstract

Regulation of microtubule dynamics depends on stochastic balance between polymerization and severing process which lead to differential spatiotemporal abundance and distribution of microtubules during cell development, differentiation, and morphogenesis. Microtubule severing by a conserved AAA family protein Katanin has emerged as an important microtubule architecture modulating process in cellular functions like division, migration, shaping and so on. Regulated by several factors, Katanin manifests connective crosstalks in network motifs in regulation of anisotropic severing pattern of microtubule protofilaments in cell type and stage dependent way. Mechanisms of structural disintegration of microtubules by Katanin involve heterogeneous mechanochemical processes and sensitivity of microtubules to Katanin plays significant roles in mitosis/meiosis, neurogenesis, cilia/flagella formation, cell wall development and so on. Deregulated and uncoordinated expression of Katanin has been shown to have implications in pathophysiological conditions. In this paper, we highlight mechanistic models and regulations of microtubule severing by Katanin in context of structure and various functions of Katanin in different organisms.

## 1. Introduction

Microtubule number and distribution in cellular cytoskeleton arrangement is important in organismal development, stage specification, shape determination and division. Microtubules, being heteropolymer of two tubulin proteins, *α*-tubulin, and *β*-tubulin, show varying degree of polymerization to maintain proper transport of intracellular cargo, divisional chromosome arrangement/segregation, and various other cellular functions. Regulation of microtubule length and spatial structural organization not only depend on nucleation and treadmilling caused tubulin polymerization, but also on regulated degree of microtubule severing. Microtubule severing requires internal cleavage in microtubule lattice followed by continuous depolymerization of tubulin dimers from ends. Microtubules are severed by enzymes like Katanin, Spastin, and Fidgetin (Table 1). Works in last two decades reveal potential role of Katanin in dynamic alteration of microtubule structure and orientation in maintenance of cellular homeostasis. Katanin is ubiquitously expressed in plants and lower to higher animals and it systemic or cell specific activity modulates differential formation and organization of microtubule arrays in cell. Physiological functioning of Katanin and the resulting microtubule fragmentation is now reported to be important underlying mechanism in cell division, neuron development, cell migration, and locomotory organelle formation. Systems network motifs involving Katanin are specified by various regulatory proteins, degradosome machineries, and coordinated functioning of other microtubule severing proteins, and these networks precisely act as effectors and stabilizing remodelers of cytoskeleton structure. Regulation of Katanin localization, interaction with other proteins influence optimality of microtubule severing and it important to understand models, regulations, and biological functions of microtubule severing by enzymes like Katanin to specify their roles in physiological and pathological conditions. 

## 2. Structure

**Figure 1:**
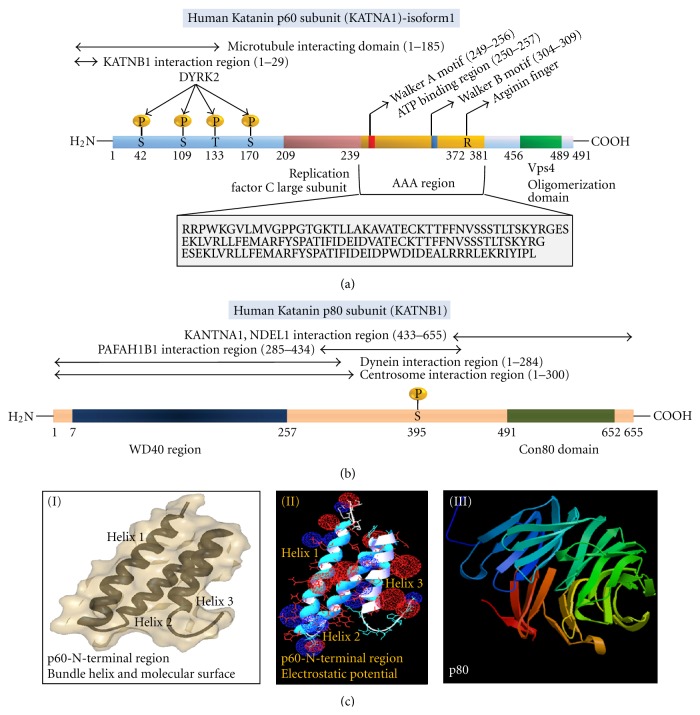
Katanin protein subunits. (a) Human Katanin p60 subunit (KATNA1). Domains in p60 include replication factor C large subunit, AAA region, Vps4 domain. AAA region contains ATP binding Walker motifs (Walker A and Walker B). N-terminal region contains KATNB1 (p80) and microtubule interaction regions. Several serine and threonine residues undergo phosphorylation by Kinases like DYRK-2. Sequence in box represents sequence of AAA region. (b) Human Katanin p80 subunit (KATNB1). WD40 and Con80 domains are parts of KATNB1. KATNB1 interacts with dynein, PAFAH1B1, KATNA1, NDEL1, and centrosome. KATNB1 has single phosphorylation site at Serine395. (c) (I) Tertiary structure of N-terminal region (residues 1–72) of p60 of mouse (*Mus musculus*) Katanin (PDB Accession Code: 2RPA). The structure shows three alpha helical strands (bundle helix) and surface of this region of protein. (II) Electrostatic potential map of N-terminal region of Mouse p60 subunit of Katanin. (Map potential to surface: red = −1.8, white = 0, blue = +1.8, dielectric constant of solvent = 80 D, and solvent ionic strength = 0). (III) Predicted tertiary structure of human KATNB1 (structure predicted using protein structure prediction server). (Acknowledgement: Protein Data Bank, Molecular Bioinformatics Center of National Chiao Tung University).

Katanin is a heterodimeric hydrolase belonging to AAA family protein (MSP1/Katanin/Spastin group) (Table 2) [1]. The two constituent subunits of Katanin are named according to their molecular weight. The smaller and catalytic subunit is 60 KDa and it is named as p60, while the larger and regulatory subunit is 80 KDa and named as p80 [2]. Human Katanin p60 subunit (KATNA1) is a 491 aminoacid long protein, which contains the AAA region functioning in ATP associated microtubule severing activity (Figure 1(a)). The AAA region contains a conserved P-loop NTPase fold which is formed by conserved aromatic aminoacids [1]. The P-loop harvests energy released from ATP hydrolysis to unfold and remodel complex tertiary structures of tubulin proteins in microtubules. The two distinct “Walker-A” and “Walker-B” motifs in p60 subunit help in ATP binding. The N-terminal residues in the Walker motifs of AAA domain participate in imparting positional stability of ATP within the protein and the residues in the C-terminal end of AAA domain help in catalyzing hydrolysis of ATP to ADP with concomitant energy release. C-terminal Vps-4 region in p60 allows this subunit to undergo polymerization [3]. 14–16 nm ring shaped unstable oligomers of p60 are reported [4]. Though the exact stoichiometry and mechanism of oligomerization is not well understood, it is observed that ADP bound p60 moieties are usually monomeric and exchange of ADP by ATP induces oligomerization of p60 monomers to hexameric state. The ATP bound hexameric p60 shows high microtubule severing activity and this activity is substantially increased in low tubulin to p60 ratio. Stability of p60 oligomers in vivo condition is reported to be sufficiently low. Labile p60 oligomers undergo spontaneous dissociation into monomers even in presence of ATP [4]. Though exact spatiotemporal regulation and kinetics of association-dissociation of p60 is not fully studied, it is documented that hexamerization happens in concerted and template dependent manner on microtubule lattice. Multiple interactions of p60 with tubulin dimers enhance multimerization process. The N-terminal region of p60 is responsible for interprotein interactions. This region can stably bind to microtubules, p80 under different concentration and signal-dependent circumstances. The crystal structure of this N-terminal region of p60 is resolved recently [3] and it depicts a three-strand helix bundle structure which is similar to microtubule interacting and trafficking domain (MIT domain) and is regarded as tubulin binding domain (TBD) (Figure 1(c)) [3]. The helix-2 and helix-3 of this structural motif have high number of positively charged aminoacids aligned at the surface. High positive charge densities in the helix surfaces allow this N-terminal region to bind negatively charged aminoacids of tubulins by electrostatic interactions. Helix-2 also contains a Ca^+2^ binding site which dramatically reduces the rate of ATP hydrolysis in p60 without interfering p60 subunit's ability to bind microtubules [5]. Binding of microtubules and C-terminal domain of p80 to NTD of p60 enhances severing activity of p60 and Ca^+2^ binding to p60 NTD brings back the Katanin activity to ground level. Stability and functional expression of p60 subunit are highly regulated by posttranslational modifications, like phosphorylation of serine and threonine aminoacids by protein kinase (like DYRK-2).

Human Katanin p80 (KATNB-1) is 655 aminoacid long protein (Figure 1(b)) which plays important role in regulation of Katanin localization, targeting and functioning of Katanin protein [6]. N-terminus of this subunit contains a WD40 domain which functions as negative regulator of microtubule disassembly at spindle poles [7]. This region also controls interaction of p80 with centrosome and other microtubule associated proteins like dynein. The C-terminal region of this protein contains Con-80 domain which mediates binding of p80 with proteins like PAFAH1B1, NDEL1 and p60. In p80, a single phosphorylation at Ser-395 has been elucidated, but its role in cellular physiology is not well emphasized yet.

## 3. Models of Microtubule Severing by Katanin

**Figure 2:**
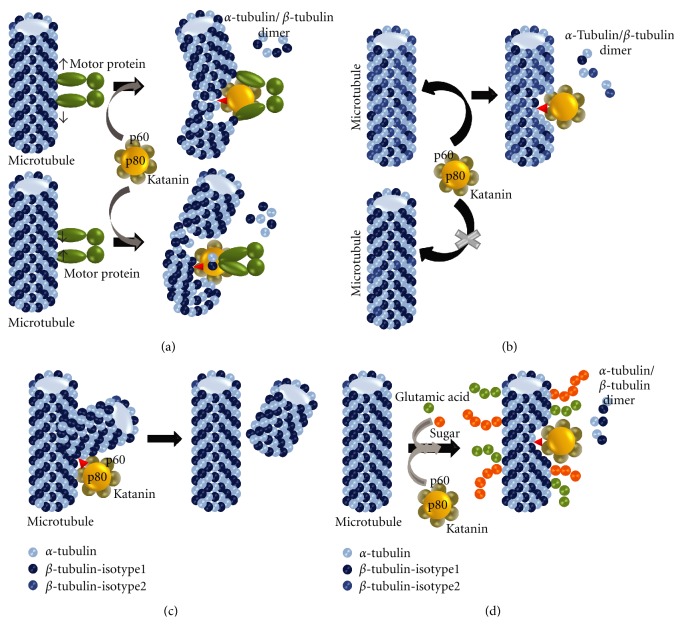
Models of Microtubule severing by Katanin. (a) Motor proteins bind to microtubules and their movements cause structural bending/deformation (into S or V shape) of microtubules to release tubulins from deformed sites. Katanin binds to these sites and enhances the process of severing. (b) Katanin preferentially binds to microtubules which are redundant in specific isotype (isotype2/TBB2) of *β*-tubulin and catalyzes severing process in these microtubules. (c) Katanin binds to branching sites of aberrantly branched microtubule mesh to severe microtubules from branching points. (d) Katanin binding and subsequent severing of microtubules is favored when microtubules are posttranslationally modified (like poly-glycated, poly-glutamylated).

Several experimental and theoretical studies came up with various models to reveal physical and molecular mechanisms of microtubule severing by enzymes like Katanin, Spastin, and so forth. A study emphasizes the role of molecular motors in development of internal exciting forces within microtubules which results in bending of microtubules into “S” and “V” shapes and these shape fluctuations cause release of tubulin dimers from deformed sites [8]. Tubulin vacant regions serve as perfect sites for Katanin deposition and initiation of severing phenomenon. Deformed spatial conformation and elastic properties of microtubules result from lattice strains generated by activity of microtubule-associated motor proteins [9]. Katanin not only binds to destabilized microtubule structures, but it also uses chemical energy of ATP hydrolysis and generates mechanical force to increase the lattice strain even more [10] (Figure 2(a)). This phenomenon represents continuous displacement of tubulin dimers from microtubules and Katanin shows fairly high processivity in removing tubulins from tubulin polymers. A second model of Katanin-mediated microtubule severing includes dynamic instability mechanism and a phenomenon which can be named as “size dependent mobilization”. Dynamic instability model explains spontaneous nucleation and/or depolymerization of microtubules in regulation of its length, shape, and form [11]. Size dependent mobilization model describes greater intracellular flux of shorter microtubules like ones which are generated from Katanin-mediated severing [12]. Apart from causing continuous depolymerization from ends, Katanin can also induce fragmentation of microtubules from defective internal regions [13] and the concept of easy transport of such fragments remains in their ability to move easily and concertedly with less exerted forces by motor proteins, like dynein [14]. Severed by Katanin, shorter microtubules are rapidly transported to various parts of the cells and maintain structural organization at those regions. Physiological need of shorter microtubules and equilibrium of local longer to shorter microtubules might also favor microtubule severing by Katanin. 

A study in *Caenorhabditis elegans* showed that Katanin binding activity is preferentially initiated at *β*-tubulins. Among the two isotypes of *β*-tubulins, Katanin shows enhanced preference for TBB-2 compared to TBB-1 [15]. Differential redundancy of these two isoforms in microtubules may regulate severing activity of Katanin (Figure 2(b)). Another study in *Drosophila melanogaster *shows a Myosin-XV homolog, Sisyphus, binds Katanin p60 subunit by its FERM domain and transports it to cellular regions, like filopodia formation sites, where microtubule reorganization requirement is critical [16].

Microtubule severing is also higher in mesh like microtubule structure compared to array like structures. Severing at microtubule cross-over provides a mechanism to remove aberrantly aligned and improperly branched microtubules (Figure 2(c)) [17]. As the angle of branching is important, a certain limit and range of angulations determine specificity and optimum activity of Katanin at branching points. Covalent Post translational modifications of tubulins, like poly-glycation, polyglutamylation, may also play important and determining functions in severing [18]. Older microtubules, having higher amount of such chemical modifications, are hotspots for Katanin binding and severing activity (Figure 2(d)) [19]. Acetylation of microtubules is also linked to increased severing activity by Katanin [20]. Deacetylation of microtubules by promiscuous activity of HDAC6 has been shown to effectively reduce microtubule depolymerization by Katanin [21].

Many theoretical studies have also enlightened mechanistic sides of Katanin-mediated microtubule severing. Based on the fundamental microtubule instability theory, a theoretical study shows possibility of random microtubule severing by Katanin. The overall microtubule numbers are, however, independent of severing rate and mean length of microtubules decrease in response to severing. The rate of severing is predicted to be proportional to microtubule length. Longer microtubules are severed to shorter microtubules of varying length and distribution [22]. Both of these results inculcate with observed cellular phenomenon of microtubule number conservation and compacted organization by short microtubule arrays. Another theoretical study using “Monte Carlo Simulation” shows that lattice defects and tensile constraints along microtubule length serve as Katanin activity sites [10]. Microtubule bending increases elastic energy in tubulin dimers to a large extent (~40 fold) and results in higher breaking and severing rates at these sites [23]. A Drosophila study experimentally establishes that lattice defects and proto-filament shift defects are primary sites for Katanin p60 binding [13]. Katanin, at those regions, stimulate microtubule severing in p60 concentration and ATP dependent manner. 

In a study conducted with both human and mouse Katanin, it's showed that helix-2 and helix-3 of the N-terminal domain (NTD) in p60 of Katanin bind to solvent accessible helix-11 (residues 386–396) and helix-12 (residues 420–430) of tubulin. p60-microtubule interactions have similarities with Vps4-ESCRT III interaction. p60 pulls down *α*-*β* tubulin dimers by providing the NTD as adaptor for microtubules. This proposed model emphasizes on formation of p60 NTD and tubulin tetramer. Specifically, in helix-3 of p60 NTD, Arg49, Gln53, Lys64, and Lys67 aminoacid residues play important part in interacting with tubulins [3, 5]. 

Kinetically, Katanin-mediated severing shows two distinct temporal phases; an initial stage of Katanin independent severing stage which increases microtubule concentration followed by Katanin dependent microtubule dissociation phase that does not increase microtubule density [11]. 

## 4. Regulation of Katanin Stability and Activity

**Figure 3:**
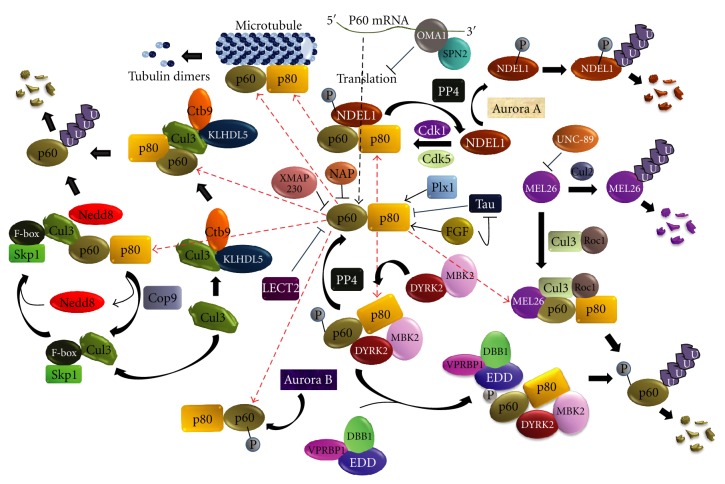
Regulatory network of Katanin protein. Major regulations of Katanin involve cell and stage specific polyubiquitination-mediated proteasomal degradation and covalent chemical modifications (like phosphorylation) at specific aminoacid residues. Different proteins interact with Katanin and direct its activation or inactivation in specific pathway. Katanin interacting proteins cause specific localization of Katanin to specific cellular regions or mark them for modifications and/or degradation.

**Figure 4:**
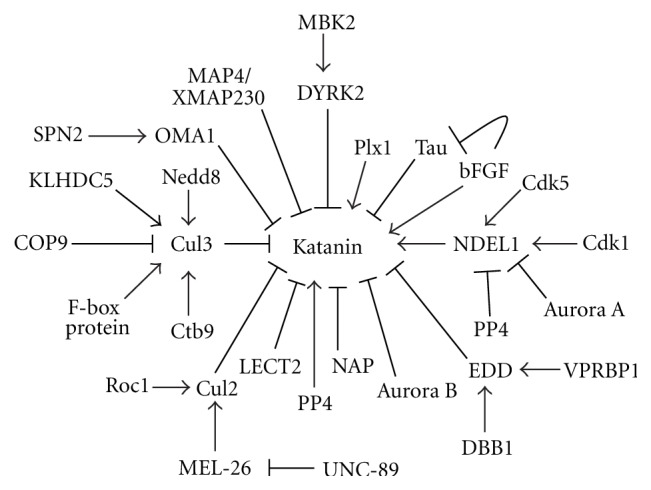
Regulators of Katanin.

Katanin stability and activity is spatiotemporally regulated by complex levels of chemical modifications, differential degradation mechanisms (Figure 3), and several Katanin interacting/regulatory proteins (Figure 4). In *C. elegans*, the major pathway of Katanin protein regulation during embryogenesis associated mitosis involves Cullin dependent degradation of Katanin. There are mainly two pathways of MEI-I (p60) degradation. During mitosis, an adaptor protein, MEL26 recruits MEI-I to CUL3-based E3 Ubiquitin Ligase and signals MEI-I to undergo polyubiquitination and subsequent proteasomal degradation [24]. BTB domain of MEL26 binds to one conserved domain of CUL3 and ROC1 helps in recruitment of MEI-I to CUL3/MEL26 complex [25]. However, during meiosis, CUL2-based Ubiquitin Ligase helps in polyubiquitination of MEL26 for it is proteasomal degradation [26]. This keeps the MEL26 level low and regulates the Katanin activity during meiotic divisions. Obscurin (UNC-89) protein can also physically interact with MEL26 protein in CUL3-MEL26 complex to diminish MEL26 protein's inhibitory activity against MEI-I [27]. In a second mechanism, Ctb9/KLHDC5 complex is reported to bind CUL3 and the final complex, CUL3/Ctb9/KLHDC,5 binds Katanin to mark it for proteasomal degradation [28]. Another CUL3 complex, SKP1/CUL3/F-Box (SCF complex), has been shown to bind Nedd8 protein and this neddylation increases affinity of E2 Ligase for E3 complex, helping the E3 complex to polyubiquitinates MEI-I for 26S proteasomal degradation [29]. An upstream regulator of this pathway, COP9, helps in deneddylation of CUL3 to passively regulate stability of MEI-I during mitosis [30]. 

Chemical modifications, like phosphorylation, have significant role in Katanin stability and activity. Protein Kinase DYRK2 can phosphorylate several serine and threonine residues of both subunits of Katanin to enhance their degradation. DYRK2 also acts as a scaffolding protein for EDVP complex (an E3 Ubiquitin Ligase containing EDD, DDB1, VPRBP) and this function of DYRK2 might have roles in facilitating Ubiquitin tagging to phosphorylated Katanin (EDD helps in ubiquitination of phospho-katanin) [31]. Just after mitosis exit, MBK2/DYRK2 Kinase complex promotes phosphorylation dependent degradation of Katanin [32, 33] with the help of anaphase promoting complex (APC) [34]. Loss of function mutation of *mbk2* shows incoherent distribution of p-granules, impaired mitosis, and disorganized microtubule arrays [33]. 

Cdk proteins, like Cdk1 and Cdk5, can phosphorylate NDEL1 and facilitate NDEL1 association with p60 subunit of Katanin [35, 36]. NDEL1 interaction with Katanin causes more deposition of Katanin to centrosome during cell division [35]. Katanin localization to centrosome is also favored by other proteins like TACC (TACC3) [37]. NDEL1 dephosphorylation is catalyzed by a serine/threonine phosphatase, Protein Phosphatase 4 (PP4), and the cycle of phosphorylation-dephosphorylation of NDEL1 is presumed to regulate Katanin activity during various stages of cell cycle [36]. PP4 can also dephosphorylate Katanin and activate it in accordance to upstream signals [38]. Phosphorylation of NDEL1 (at Ser251) by Aurora-A also helps polyubiquitin mediated proteasomal degradation of NDEL1 and facilitate release of Katanin p60 from centrosomes [37]. Specific aminoacid residues in Katanin subunits of different species and their differential modifications may play determining role in Katanin activity. P60 of *Xenopus laevis *differs from *Xenopus tropicalis* in a key Ser131 residue which can be phosphorylated by Aurora-B. Phosphorylation of this p60-Ser131 dramatically reduces severing activity of Katanin in *X. laevis* meiotic egg extracts [39]. 

In Xenopus egg extracts, a MAP4 homologous protein XMAP230 shows inhibitory activity against Katanin [40]. However, Cyclin-B/Cdk1 complex can reverse this inhibitory effect. Another protein resembling Polo-like kinase, Plx1, acts as coactivator of Katanin to increase its activity in terms of microtubule severing [40]. 

A single report suggests that *C. elegans* Katanin p60 homolog, MEI-1, can be regulated at translation level. SPN-2 protein binds to OMA1 and directs OMA1 to bind 3′-UTR of mei-1 mRNA. OMA1 subsequently inhibits translational expression of mei-1 mRNA during embryonic mitosis [41]. 

## 5. Role of Katanin in Cell Division

**Figure 5:**
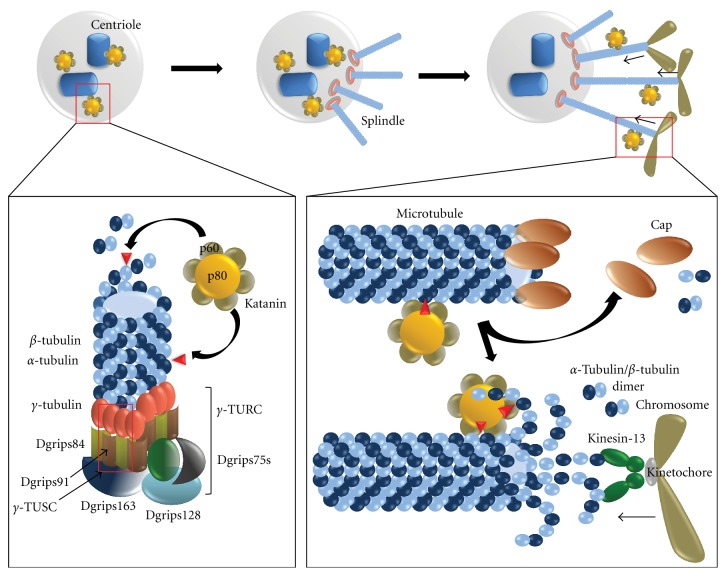
Functions of Katanin in cell division. Katanin localizes in centrosome where it severs centriolar microtubules and cleaves more *γ*-tubulins to maintain adequate distribution of *γ*-TURC complex which can serve as site for nucleation and formation of new spindle during early divisional stage. Tubulins generated from severing of microtubules by enzymes like Katanin maintain normal pool of tubulin proteins for elongation of spindle by continuous tubulin polymerization. In late divisional stages, Katanin severs microtubules near plus ends (ends holding chromosomes) and maintains chromosome movement towards spindle pole by continuous spindle shortening (Pacman Flux).

Role of Katanin in cell division is well established (Figure 5). Though initial studies indicates most of the roles of Katanin are restricted in meiotic divisions, recent studies indicate that Katanin has functional activity even in mitotic divisions [42]. Katanin, along with its functional orthologs like Spastin, Lipotransin, Fidgetin, takes active part in microtubule processing during divisional stages. Early reports in Xenopus show Katanin to be a primary protein involved in microtubule dependent regulation of spindle formation [43]. Spindle microtubules are depolymerized by Katanin and this keeps the balance in maintaining optimum K-fiber microtubule bundle number during division [39]. In *C. elegans,* Katanin activity in spindle formation is reported to be only required in meiotic divisions and not in mitotic divisions [44]. Assembly and formation of meiotic spindles from centriole poles are shown to be mediated by asymmetric deposition of Katanin at these poles. However, Katanin's severing activity is not shown to be required for such spindle formation [45]. During meiosis I, bipolar spindle move towards the cortex after formation. Katanin helps in spindle translocation towards cortex, and in Katanin depleted oocytes, FZY1/CDC20 complex, and cytoplasmic streaming compensates activity of Katanin to allow movement of unstable spindle towards cortex [46].

In *Drosophila melanogaster*, Pacman Flux during anaphase chromosome segregation from metaphase plate towards spindle pole is governed by activity of Katanin [47]. Katanin severs microtubules near plus ends and helps depolymerization of microtubules near the microtubule-chromosome attachment site for continued movement of chromosomes towards spindle pole in late anaphase stage. Other microtubule severing enzymes like Spastin and Fidgetin remove tubulins from microtubule minus ends in pole to cause active depolymerization of microtubules at this end to fasten chromosome movement towards spindle pole. Redistribution of *γ*-tubulin ring complex from centrosome to mitotic spindles and subsequent addition of these *γ*-tubulin to ends of microtubules is thought to be largely influenced by Katanin caused centrosomal microtubule degradation [48]. To compensate the inefficient nucleation process in microtubule polymerization, large numbers of short microtubules are generated by Katanin activity near chromosomes to increase the chance and efficiency of nucleation and proper orientation of cytoskeleton [49]. A recently discovered Katanin like protein 1 (KL1) is also shown to be localized in spindle poles and alters kinetics of *γ*-tubulin association to plus ends of microtubules to modulate it is stability [50]. 

Katanin-mediated regulation of spindle assembly has now been shown to be an important mechanism in human male gametogenesis. Specifically, Katanin p80 defines the spindle structure in male meiotic divisions and also exerts it is effects at later stages when it takes active part in clearing midbody microtubules for proper progression of cytokinesis. Proper microtubule positioning by Katanin in sperms results in structural shaping of head, axoneme, tail and influences sperm motility [51]. Katanin related protein, KATNAL-1, has also been reported to be involved in spermatogenesis. It maintains exact parallel microtubule array structure in the testicular sertoli cells and this allows flux of nutrients from sertoli cells to germ cells for spermatid development and movement through seminiferous epithelium [52]. Inhibited expression of Katanin p80 and KATNAL-1 are associated with abnormal sperm production, sperm motility, and male infertility. 

## 6. Role of Katanin in Neuron Development

**Figure 6:**
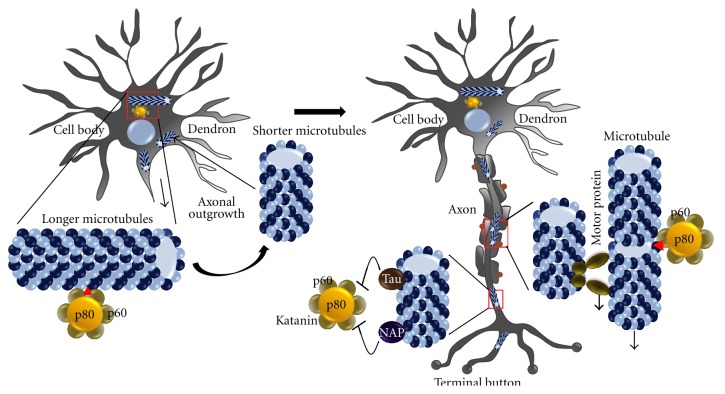
Role of Katanin in neuron development. In neuron cell body, Katanin severs longer microtubules to generate shorter microtubules which can be easily transported to axons with the help of motor proteins to help axon outgrowth and target reaching. In axons, binding of NAP and Tau proteins to shorter microtubules prevent further activity of Katanin in axonal microtubules.

In various groups of studies, Katanin has been shown to be key factor in neuronal development in terms of formation and maintenance of protrusions like axons (Figure 6) [53]. Katanin helps in axonal outgrowth by regulating and optimizing microtubule polymerization dynamics [54]. The “Growth Cone Mobility Model,” which represents concerted growth of neuronal microtubules, also emphasizes the role of Katanin-mediated microtubule severing for proper arrangements of microtubules along the axon [55]. Primary development of hippocampal neurons and neurite extensions are facilitated by Leukocyte cell derived chemotaxin 2 (LECT2) factor. It is reported that LECT2 functions in repression of Katanin p60 to regulate microtubular morphallaxis [56]. During formation of interstitial axonal branches, Katanin severed shorter microtubules with serve as sites for new branching and microtubule growth. Basic fibroblast growth factor (bFGF) is reported to upregulate expression of Katanin [57]. It also phosphorylates Tau protein to release them from axonal microtubules. Upregulated Katanin and Tau depleted microtubules enhance microtubule severing and increase the rate and frequency of axonal microtubule branching [58]. In axonal development, activity of Katanin is highly coordinated with localization and activity of another microtubule severing enzyme, Spastin [59]. Spastin is usually located at axonal branch sites where it modulates longer microtubule rearrangement whereas Katanin severs microtubules in protruded axons to make branched and intermingled microtubule rearrangement. IGF1 has been shown to induce different responses for Katanin and Spastin [60]. While Katanin is insensitive to IGF1, Spastin caused microtubule process lengths and process numbers is reduced in response to IGF1 response. Though the entire mechanism of preferential localization of Spastin and Katanin is not known, it is believed that cooperative and synergistic functioning in between them is essential for proper microtubule organization pattern in axons. Centrosome in neurons serves as site for microtubule nucleation and concentrated localization of Katanin at centrosome helps in release of microtubules from centrosome [54]. Katanin remains dispersed in normal neuronal cell body where it severs microtubules which are released from centrosome. This severing of released microtubules is to process them into even shorter microtubules that can be transported to axons to allow axonal outgrowth. Distribution and concentration of two subunits of Katanin also varies during different developmental stages of neuron [61]. Intense p60 expression is seen in specific differentiation stages. It is more abundant in axonal length and tips to enhance the total number of microtubule processes. On the contrary, fluctuation of p80 expression is less during neuron development and a large fraction of p80 is localized in cell body to play limiting role in altering microtubule mass. The mechanism of regulation of this asymmetric distribution of p60 and p80 in neurons is not known, but this certainly causes more differentiation in severing pattern of microtubules along axons compared to cell body. This differentiation is extreme when axons reach their targets. Not only Katanin, but sensitivity of microtubules is presumed to be influenced by other less known microtubule severing influencing factors. 

Though Katanin activity is restricted in dendrites, dendrite pruning in *Drosophila melanogaster* sensory neurons involves noticeable degree of Katanin p60 like-1-(Kat-60L1-) mediated microtubule severing [62]. Katanin levels are also high in proximal dendrite regions of these sensory neurons and show higher degree of severing in these regions [62]. Specificity of Katanin to axonal microtubules compared to dendrite microtubules depends on both acetylation status and Tau protein association to microtubules [21]. Stretches of microtubules in axons are more acetylated and these regions show higher sensitivity to Katanin (but not for Spastin) than relatively less acetylated dendrite microtubules. Katanin p60 like protein-1 (Kat-p60L1) regulates *Drosophila* larval class IV dendritic neuron's dendrite morphology. This protein acts in microtubule growth for formation of filopodia like structures to help in stabilizing terminal dendritic structure during dendritic arbor development [63]. 

## 7. Role of Katanin in Disease Modulation

Role of Katanin in disease induction, progression, differentiation, and stage determination is not studied intensively. Few studies are revealing altered Katanin functioning in modulation of diseases. Expression and functional pattern of Katanin p60 subunit in prostate cancer show variability when compared to normal prostate [64]. Higher expression of p60 in prostate cancer causes inhibition of cell proliferation but allows cancerous cells to take more aggressive migration property. In normal prostates, expression of p60 is only limited in basal cells while in prostate adenocarcinoma cells, p60 expression is much higher throughout prostate. Role of Katanin in metastasis is presumed to be mediated by enhanced microtubule severing activity which enforces reorganization of cytoskeleton structure to help forming cellular protrusions involved in cell motility [64, 65]. Higher p60 expression is also observed in bone metastatic cells. Study in *Drosophila melanogaster* shows that Katanin (*Dm*-Kat60) regulates depolymerization of tubulins from plus ends of microtubules in cortex of interphase cells and this phenomenon highly regulates cellular attachments and migration [66]. A tumor suppressor protein, LAPSER1/LZTS2 (LAPSER1) can bind to regulatory p80 subunit of Katanin at centrosome to regulate cytokinesis of cell division by modulating central spindle formation [67]. Regulation of p80 by LAPSER1 also limits migration of metastatic prostate cancer cells by enhancing *γ*-tubulin dissipation from microtubule end [68]. Loss of *γ*-tubulin from microtubule ends destabilizes microtubules, finally leading to catastrophe in microtubule organization in mobility associated protrusions. Loss of heterozygosity of LAPSER1 gene correlates with increased Katanin activity and favors metastasis. Again, overexpression of LAPSER1 causes aberrant mitosis followed by failure in cytokinesis due to deregulated activity of Katanin p80 subunit. 

In Tauopathies like Alzheimer's disease, elevated Katanin expression causes extensive degradation of Tau protein depleted microtubules in neurons [12, 58]. Activity of Katanin in neurons is dependent upon whether microtubule is associated with Tau protein. Tau- and microtubule-associated protein 2 (MAP-2) have similar microtubule binding domains and they bind to microtubules to protect them from severing by Katanin [58]. Tau released from microtubules is caused by it is phosphorylation and this causes uncoordinated fragmentation of microtubules [57]. Acetylation of tubulins also stimulates Katanin-mediated microtubule severing [21]. HDAC catalyzed deacetylation of tubulins and/or binding of peptides (like NAP) to microtubules efficiently prevent Katanin activity [20]. NAP (Davunetide) is an ADNP protein-derived eight-aminoacid long small neuropeptide which binds to Tau lost faces of microtubules and shields these faces from Katanin binding [69]. Increased NAP functions show positive responses in schizophrenic patients and mitigate with decreased microtubule severing by Katanin [69]. This phenomenon leads to reduced level of neuroapoptosis. Interplay of Tau binding and covalent chemical modifications in microtubules are supposed to be key regulatory factors in optimizing Katanin activity during neuronal outgrowth in neuropathies [18].

Mutations in AAA proteins like Spastin and Katanin are seen to be associated with Hereditary Spastic Paraplegia (HSP) [70]. This disease manifests abnormal cellular microtubule number, distribution, arrangement, and this may be attributed to reduced or aberrant functioning of mutated Katanin protein. Like many other MAPs, Katanin contains a sequence motif, LisH, which has implications in diseases like Miller-Dieker lissencephaly syndrome, Treacher Collins syndrome, Oral-facial-digital type I syndrome, Ocular albinism, and the role of Katanin in these diseases is still obscure and seeks further investigations [71].

## 8. Katanin Functions in Plant and ****Lower Organisms

### 8.1. Role in Plants

A considerable number of studies have been conducted in *Arabidopsis thaliana* to understand various cellular and physiological roles of Katanin in plant systems. Like in animals, one major role of Katanin in plants is to regulate cell division by regulating spindle microtubule severing [72]. Katanin mutated *Arabidopsis thaliana* cell lines, like *fra2* mutant and *leu1* mutant, show abnormal microtubule orientation, alignment, and polarity in divisional phases. These defects in organization of microtubule arrays finally lead to altered division plane orientation.

Katanin plays important role in cortical microtubule organization in both dividing and differentiated cells to maintain plant shape. Forceful reorientation of plant shape like transformation of longitudinal growth to lateral growth by chemicals (e.g., 1-amino cyclopropane-1-carboxylic acid [ACC]) correlates with upregulated Katanin function and subsequent changes in microtubule bundle direction [73]. *Arabidopsis thaliana* lines, which show constitutive higher expression of Katanin, also show similar results in shortening and thickening of cortical microtubules [74]. Redistribution of Katanin severed shorter microtubules in cortical regions depends upon generation of motile microtubules which possess enhanced ability of being assembled into bundles. New microtubule formation is also maintained by branching from existing microtubules followed by elongation with tubulin polymerization. The *γ*-tubulin complex's constituent proteins, GCP2 and GCP3 are deposited on the surface of previously formed microtubules and make platform for nucleation of new microtubules [49]. Though this lateral nucleation and microtubule formation are speculated to be highly regulated by Katanin, the mechanism and associative interplay of Katanin and other proteins are not completely delineated. In tobacco plants, MAP65-1c protein is shown to bind microtubules and protect them from Katanin [75].

A very recent study indicates that growth in plant tissue is not homogeneous. The heterogeneity in growth is a response to differential mechanical forces imparted on growing neighboring cells and Katanin-mediated severing of microtubules plays important role in such heterogeneity and morphogenesis [76]. *Arabidopsis thaliana* Katanin mutant, *atktn1*, shows loss of microtubule arrangement and associated growth pattern diversity due to its lost ability in perceiving mechanical forces that balance homeostasis in cell growth. 

Leaf senescence involves increased and early degradation of microtubule network in epidermis cells of mesophyll tissue. Dark plant leaves repress Katanin and it's homologue and these leaves also undergo less senescence. It's presumed that Katanin helps in degradation of microtubules in leaf cells to fasten leaf senescence process in response to light and other environmental cues [77]. 

Microtubule severing by Katanin is responsive to plant hormones like ethylene, gibberellin [78]. Hormonal induction of Katanin finally determines microfibril content in cell wall. Katanin mutant in *Arabidopsis thaliana* (*leu1*) produces truncated Katanin with diminished activity and this mutant shows severe defects in cell wall formation [78]. *fra2* mutant also shows aberrant orientation and deposition of cellulose and hemicellulose microfibrils and increase of lignin content [79]. Beside of being disorganized and differentially directed towards various directions, these microfibril bands show reduced primary and secondary cell wall elongation. Katanin allows constructive and proper organization of microfibrils to maintain cell wall's cellulosic sheath formation.

In rice plants, Katanin p60 homologous protein, DGL1, is reported to be involved in cell elongation and duplication. This protein is also seen to be causing upregulation of gibberellin biosynthesis genes without interfering in this hormone's signaling cascade [80]. Katanin has been recently shown to be involved in production of fibrous tissues in plants. In common cotton plant (*Gossypium spp.*), Katanin helps in formation of longer cotton fibers and maintains proper seed oil to endosperm oil ration [81]. 

Specification of root epidermis cells from meristematic cells occurs by regulated differentiation driven by Katanin-mediated microtubule severing. Severed microtubules dictate positional cues during root epidermal cell development [82].

### 8.2. Role in Lower Organisms

Other than functions in cell division, Katanin is shown to be involved in structural formation, maintenance, and functioning of locomotory organelles like flagella and cilia in lower organisms. In Trypanosomatid parasites (*Leishmania major*, *Trypanosoma brucei*, etc.), Katanin p60 reduces length of flagella and p80 regulates cytoskeleton structure [83]. In Chlamydomonas, Katanin helps in cell cycle responsive flagellar resorption by active severing of flagella at transition zones to cause release of doublet microtubules (ciliary disassembly) from basal bodies [84]. Accumulation and microtubule severing by Katanin at flagellar basal bodies is Ca^+2^ signal dependent [85]. Maintenance of doublet central apparatus of flagella is also dependent on proper expression of Katanin p80 homolog PF15 [86]. In Tetrahymena, activity of Katanin reduces mass and stability of microtubules in cilia [19]. Tubulins generated by Katanin activity eventually aid in increasing ciliary tubulin concentration for spatial regulation of microtubule formation. Proper division of *Trypanosoma brucei *in its parasitic stage in human blood requires activity of Katanin. Early stage cytokinesis of the organism is helped by KAT60 while the later furrow formation stage is supported by KAT80 [87]. 

## 9. Concluding Remarks

Severing of microtubules introduces itself as an important cytoskeleton reinforcing process that operates at multitier levels during developmental and differentiation stages. Since it is discovery as microtubule severing enzyme, the role of Katanin has been appreciated in coordinating cellular homeostasis. Regulation of microtubule dynamics by it is differential structural stabilization and destabilization by Katanin reveals an important aspect of addressing systemic and/or cell/stage-specific intricate microtubule modulations which finally lead to specification of various cellular processes. Our review summarizes various models of microtubule severing by Katanin, regulations of Katanin in systems network, and representation of major cellular functions of Katanin. Though flourishing researches have been conducted to understand molecular mechanisms of Katanin-mediated microtubule severing and it is consequences in cell, tissue and organismal level; review of researches and literatures on Katanin led us to speculate multiple questions which can inspire progressive research. We summarize few of such questions.

1. Is Katanin expression regulated at transcription level? As Katanin expression varies in cell and developmental stage-dependent way, it is inspiring to understand transcriptional regulation of Katanin.
2. What is the entire biochemical and molecular mechanism of microtubule severing by Katanin? Though structure of N-terminal region of p60 subunit of Katanin is solved, crystal structure of full p60 can give mechanistic insights of severing in terms of catalytic cleft, residues and processes.
3. What is the role of Katanin in neurons, stem cells, and cancer cells in terms of proliferation and developmental potential?
4. What is the role of Katanin-induced microtubule shortening in alteration of invasive property of cancer cell? Cytoskeleton alteration is a major aspect of malignant cancerous cells and studies on role of Katanin in invasion can open new dimensions of controlling metastasis.

As new discoveries are revealing new information on Katanin-based microtubule structure and organization alteration, we are in the brink of starting a voyage of understanding chemical, biophysical, and biological functions of microtubule severing by enzymes like Katanin, Spastin, and so forth.

## Figures and Tables

**Table 1:** Katanin and other different AAA family microtubule severing proteins.

Protein	Species	Length∗	Domains and length∗	NCBI accession number
Katanin	*Homo sapiens*	p60–491 p80–655	p60-PRK04195 (209–265), AAA (241–382), Vps4_C (456–489), p80-WD40 (7–257), Con80 (492–652)	p60: AAC25114 p80: NP_005877
Spastin	*Homo sapiens*	584	MIT (116–195), AAA (313–476)	AAI50261
Fidgetin	*Homo sapiens*	759	AAA (519–654), Vps4_C (707–756)	NP_060556
Lipotransin	*Mus musculus*	491	PRK04195 (211–265) AAA (241–382) Vps4_C (456–489)	AAD42087
Katanin-like 1 (KL1)	*Homo sapiens*	490	AAA (211–380) Vps4_C (447–488)	NP_001014402
Fidgetin-like 1	*Homo sapiens*	674	AAA (531–567) Vps4_C (631–671)	AAH51867
MSP1	*Saccharomyces cerevisiae*	362	VWA (10–164) AAA (123–257)	CAA97015
VPS4A	*Homo sapiens*	437	MIT_VPS4 (4–79) AAA (130–293) Vps4_C (373–434)	NP_037377

∗ All lengths are given in amino acid stretch length in the polypeptide/protein.

(Domain Abbreviations: PRK04195: replication factor C-large subunit, Vps4_C: Vps4 C-terminal oligomerization domain, WD40: beta transducin repeat of 40 aminoacids ending with Tryptophan and Aspartate, MIT: microtubule interacting and trafficking domain, AAA: ATPase associated with diverse cellular activities, VWA: von Willebrand factor type A domain).

**Table 2:** Katanin protein subunits p60 and p80 in different organisms.

Species	p60 length∗	p80 length∗	AAA region∗	Walker A motif∗	Walker B motif∗	NCBI accession number
*Homo sapiens*	491	655	241–382	249–256	304–309	p60: AAC25114 p80: NP_005877
*Xenopus laevis*	486	351	238– 379	246–253	301–306	p60: AAD53310 p80: AAC25113
*Arabidopsis thaliana*	523	836	271–414	279–286	334–339	p60: AEE36390 p80: AED93167
*Drosophila melanogaster*	571	823	325–462	333–340	388–393	p60: AAF34687 p80: AAF34688
*Caenorhabditis elegans*	472	280	225–363	233–240	288–293	p60: AAA28109 p80: AAF62184

∗ All lengths and regions are given in aminoacid stretch length in the polypeptide/protein.

## Abbreviations

MSP1: Merozoite-specific protein1
KATNA1: Katanin p60 (ATPase containing) subunit A1
KATNB1: Katanin p80 WD40 containing subunit B1
PAFAH1B1: Platelet-Activating factor acetyl hydrolase 1B1
NDEL1: Nuclear distribution protein nudE Like 1
TBB1: Tubulin *β*1
TBB2: Tubulin *β*2
ESCRT III: Endosomal sorting complex required for transport III
Vps4: Vacuolar protein sorting associated protein-4
MEI-I: Meiotic spindle formation protein-I
CUL3: Cullin3
CUL2: Cullin2
KLHDC5: Kelch domain containing protein-5
SKP1: S-phase associated kinase protein-1
Nedd8: Neural precursor cell expressed developmentally down-regulated protein-8
DYRK-2: Dual specific tyrosine-phosphorylation regulated kinase-2
HDAC: Histone deacetylase
DDB1: DNA damage binding protein-1
VPRBP: Vpr (HIV1) binding protein-1
TACC3: Transforming acidic coiled coil protein-3
MAP (2,4,65c): Microtubule associated protein (2,4,65c)
Cdk: Cyclin dependent kinase
Plx1: Polo like kinase-1
SPN2: Septin like protein-2
OMA1: Mitochondrial metalloendopeptidase-1
FZY1: Fizzy1
KL1: Katanin like protein1
LECT2: Leukocyte cell derived chemotaxin2
bFGF: Basic fibroblast growth factor
IGF1: Insulin like growth factor1
LAPSER1: Leucine zipper tumor suppressor protein1
NAD: Davunetide
ADNP: Activity dependent neuroprotective protein
GCP (2,3): Granulocyte chemotactic protein (2,3)
DGL1: Dolichyl diphospho-oligosaccharide protein1.
